# Putative Chemosensory Receptors of the Codling Moth, *Cydia pomonella*, Identified by Antennal Transcriptome Analysis

**DOI:** 10.1371/journal.pone.0031620

**Published:** 2012-02-20

**Authors:** Jonas M. Bengtsson, Federica Trona, Nicolas Montagné, Gianfranco Anfora, Rickard Ignell, Peter Witzgall, Emmanuelle Jacquin-Joly

**Affiliations:** 1 Research and Innovation Centre, Fondazione Edmund Mach, San Michele all'Adige, Italy; 2 UMR-A 1272 - Physiologie de l'Insecte: Signalisation et Communication, INRA, Versailles, France; 3 UMR-A 1272 - Physiologie de l'Insecte: Signalisation et Communication, UPMC - Université Paris 6, Paris, France; 4 Division of Chemical Ecology, Department of Plant Protection Biology, Swedish University of Agricultural Sciences, Alnarp, Sweden; Duke University, United States of America

## Abstract

The codling moth, *Cydia pomonella*, is an important fruit pest worldwide. As nocturnal animals, adults depend to a large extent on olfactory cues for detection of food and mates, and, for females, oviposition sites. In insects, odor detection is mediated by odorant receptors (ORs) and ionotropic receptors (IRs), which ensure the specificity of the olfactory sensory neuron responses. In this study, our aim was to identify chemosensory receptors in the codling moth as a means to uncover new targets for behavioral interference. Using next-generation sequencing techniques, we identified a total of 43 candidate ORs, one gustatory receptor and 15 IRs in the antennal transcriptome. Through Blast and sequence similarity analyses we annotated the insect obligatory co-receptor ORco, five genes clustering in a conserved clade containing sex pheromone receptors, one homolog of the *Bombyx mori* female-enriched receptor BmorOR30 (but no homologs of the other *B. mori* female-enriched receptors) and one gene clustering in the sugar receptor family. Among the candidate IRs, we identified homologs of the two highly conserved co-receptors IR8a and IR25a, and one homolog of an IR involved in phenylethyl amine detection in *Drosophila*. Our results open for functional characterization of the chemosensory receptors of *C. pomonella*, with potential for new or refined applications of semiochemicals for control of this pest insect.

## Introduction

Insects employ olfaction for several vital tasks, such as the search for food and mates, and location of suitable oviposition sites by females [1]. Volatile compounds are detected by olfactory sensory neurons (OSNs) which are present on antennae and palps. Several families of transmembrane proteins appear to form binding sites for odorant molecules at the membrane surface of OSNs, of which the odorant receptor (OR) family is the most widely expressed [2]. OR proteins of insects have seven transmembrane domains, but have the N-terminus on the inside of the cell membrane, i.e. an inverted topology compared to vertebrate ORs, to which they are unrelated [3]. To function, they require the presence of a conserved co-receptor named ORco [3], [4]. Subsets of OSNs also express proteins from the gustatory receptor (GR) family [5], which are structurally related to ORs, or ionotropic receptors (IRs), which are related to ionotropic glutamate receptors [6].

Insect OR genes are highly diverse, and their number varies greatly between species, with most having between 50 and 200. They represent an extreme case of birth-and-death evolution, with repeated duplication and deletion events, possibly reflecting the rapid evolution of the olfactory sense [7]. The first insect ORs were identified in *Drosophila melanogaster* by screening genomic data for genes that encoded proteins with seven transmembrane domains and increased expression in the olfactory sensory appendages, the antennae and palps [8], [9], [10]. Except for ORco orthologs that are highly conserved in insects, the low level of sequence identity (20–40%) of ORs led to homology cloning only being successful for receptors involved in pheromone detection (pheromone receptors, PRs) [11], [12], [13] and exceptionally conserved ORs [14], with most other ORs identified by genome annotation. Recently, transcriptomic approaches have been used to identify chemosensory receptors in species with no sequenced genome available. To date, high-throughput sequencing of antennal transcriptomes has been successful in identifying substantial numbers of candidate ORs in *Manduca sexta* [15] and *Spodoptera littoralis* [16].

Insect IR genes were discovered by a bioinformatic screen for insect-specific genes with enriched expression in OSNs [6]. Further wide screening of available animal genomes revealed that, unlike ORs, IRs are present across protostomia (containing arthropods, nematodes, annelids and molluscs) [17]. IRs appear to have evolved from ionotropic glutamate receptors (iGlurs), which are involved in synaptic signal transduction in both vertebrates and invertebrates. Since IRs are more conserved than ORs, it has been possible to identify several paralogous lineages among insects. Multiple IRs form functional complexes, in combinations of two or more subunits, comprising individual odor-specific receptors and one or two broadly expressed receptors (in *D. melanogaster*, IR25a and IR8a) that function as co-receptors [18]. Transcriptomic approaches aiming at identifying OR genes in insects have also been successful in IR gene identification, e.g. in *S. littoralis* [19].

The identification of ORs and IRs in pest insects is especially significant due to their potential as new targets in insect pest control. The codling moth, *Cydia pomonella* (L.) (Lepidoptera, Tortricidae), is an economically important pest on pome fruit worldwide. Control of codling moth largely relies on insecticides [20], although mating disruption has been developed as an environmentally safe alternative [21], [22]. In mating disruption, sexual communication and mate-finding is disrupted by aerial permeation of apple orchards with synthetic pheromone. The method is, however, not reliable at high population densities. There are also indications that plant compounds interact with pheromone communication – for example, ethyl (*E,Z*)-2,4-decadienoate, a pear-derived compound referred to as pear ester, can interact with the male attraction to the pheromone of *C. pomonella*, codlemone [23]. Indeed, electrophysiological work indicates that male moths possess OSNs capable of detecting both codlemone and pear ester [24]. While some short fragments of candidate ORs have been identified for *C. pomonella* [25], identification of a wider range of codling moth chemoreceptors will enable investigation into the receptor mechanisms underlying pheromone communication, the interaction between host plant volatiles and pheromone, and the identification of further plant attractants. Such attractants could have potential for behavioral manipulation of females, which are only indirectly affected by mating disruption.

In order to make OR and IR gene identification possible in an organism where a full genome is unavailable, we employed a transcriptome approach based on next-generation sequencing of antennae of both male and female *C. pomonella*. This approach appeared to be effective in identifying large sets of ORs and IRs.

## Methods

### Insects, cDNA library construction, and bioinformatics

*C. pomonella* pupae were obtained from a laboratory rearing (Andermatt Biocontrol, Grossdietwil, Switzerland), and adults were allowed to emerge in cages kept at 23°C, 70±5% RH and 16 h∶8 h light/dark cycle, and were fed 10% sugar solution. Antennae were removed at the base of the pedicel from 2–3 day old female and male insects with sharp forceps, and immediately stored at −80°C. Total RNAs from male and female antennae were extracted using TRIzol (Invitrogen, Carlsbad, CA, USA). The antennal RNAs were quantified using Nanodrop. Duplex-specific-nuclease normalized cDNA libraries were constructed (LGC GmbH, Berlin, Germany) and sequenced using next-generation sequencing (Roche 454 GS FLX Titanium, LGC GmbH, ½ Picotiter plate per sample). Short or low-quality reads and linker sequences were removed by the program seqclean (http://compbio.dfci.harvard.edu/tgi/software/). Male and female reads were assembled separately into contigs using Newbler (454 Life Sciences, Branford, US-CT).

Male and female contigs were analyzed through bioinformatics, in search of candidate ORs and IRs. Tblastn searches were performed using available amino acid sequences of Lepidoptera ORs and insect IRs. Contigs presenting similarity to chemosensory genes were further assembled using Cap3 (http://pbil.univ-lyon1.fr/cap3.php), open reading frames (ORFs) were searched and translated to amino acid sequences using ExPASy (http://www.expasy.org/), and tBlastn on the Genbank non-redundant database (http://blast.ncbi.nlm.nih.gov/Blast.cgi) was used to verify their annotation. The identity of OR and IR sequences was studied by sequence alignment using MAFFT version 6 (http://mafft.cbrc.jp/alignment/server/) [26]. Transmembrane domains were predicted for *C. pomonella* ORs and IRs deemed to be complete (based on the presence of start and stop codons, and contig length compared to similar OR sequences in other species). Three transmembrane domain prediction models were used: HMMTop (http://www.enzim.hu/hmmtop/), TMHMM 2.0 (http://www.cbs.dtu.dk/services/TMHMM/), and TMPred (http://www.ch.embnet.org/software/TMPRED_form.html).

### Sequence similarity analysis

To confirm the annotation of the candidate chemosensory receptors and to search for orthologs, putative *C. pomonella* OR and IR sequences (further defined as CpomORs and CpomIRs) were included in data sets to build neighbor-joining trees. In the OR data set, 44 protein sequences identified as candidate CpomORs were aligned with OR repertoires identified in other Lepidoptera (*Bombyx mori*, *Heliothis virescens*, *M. sexta*, and *S. littoralis*) and with the five full-length OR sequences identified in other tortricid moths (*Epiphyas postvittana*, *Planotortrix excessana* and *Ctenopseustis obliquana*). As they are structurally related to ORs and can be expressed in antennae, GR sequences identified in these species were also included in the dataset, except the 55 sequences of *B. mori* belonging to the putative bitter receptor clade. Ultimately, the OR data set contained 232 sequences.

In the IR dataset, 15 *C. pomonella* candidate IRs were added to sequences identified in *B. mori*, *M. sexta* and *S. littoralis*. Since IRs are more conserved than ORs among insects, IR sequences from non-Lepidoptera species (*Apis mellifera*, *D. melanogaster*, and *Tribolium castaneum*) were also included in the data set. In addition, *D. melanogaster* iGluR sequences were included, and the final data set contained 159 sequences.

Sequences were aligned using MAFFT , using the FFT-NS-2 algorithm and default parameters. Unrooted neighbor-joining trees were constructed using the BioNJ algorithm and Poisson correction of distances, as implemented in Seaview v.4 [27]. Trees were drawn with iTOL [28]. *C. pomonella* chemosensory genes were numbered according to their closest homologs in sequence similarity analyses.

### Reverse Transcription PCR for expression analysis

To verify expression of the putative ORs identified from the transcriptome and to study differential expression between the sexes, RT-PCR was performed using cDNAs prepared from male and female antennae. RNAs were extracted as described above, treated with DNAse (RQ1, Promega, Madison, WI, USA) and corresponding cDNAs were synthesized using the RT-for-PCR kit (Clontech, Mountain View, CA, USA) following the recommended protocol. Testing was restricted to contigs which were of sufficient length to enable the construction of primers giving a product of 300 bp or more. Primers were designed manually, or using the Primer3 tool (http://frodo.wi.mit.edu/primer3/) and sequences are available in Table 1. RedTaq (Sigma Aldrich, St Louis, MO, USA) was used for PCR reactions, which consisted of an initial 5-minute step at 94°C, and then 35 cycles of 94°C for 1 min, 55, 58 or 63°C (depending on primers) for 2 min, and 72°C for 3 min, and a final 7-minute step at 72°C. For some amplifications, 40 cycles were used to increase the amount of product available for sequencing. Product identity was confirmed by direct sequencing, following gel extraction (QIAquick Gel Extraction Kit, Qiagen, Hilden, Germany). Each PCR reaction was repeated three times and controls consisted of no template PCRs. All PCRs were performed in parallel on a genomic DNA (gDNA) template. No amplification or amplifications of larger size products were observed in most cases, revealing that no significant gDNA contamination occurred in our cDNA preparations. Products were analyzed on a 1% agarose gel and visualized after staining with ethidium bromide using a Gel Doc XR (Bio-Rad, Hercules, CA, USA).

**Table 1:** Primers for RT-PCR expression analyses of *Cydia pomonella* ORs.

OR	Forward Primer (5′ to 3′)	Reverse primer (3′ to 5′)	Predicted Tm (°C)
1	GAGCCGGAGGCCTTGGTAA	TCTGCGAATGTGGCTAGCA	55
2	CGACAAGGAGAGCAACGATACG	TGAGACCATCGATCTTTGTCGCTT	58
3	AGATGAAGAGTATCGGAATTGCATGG	CCAACTGGGATCATGCCACAAGC	58
4	CCTCACAGGCAGTTTGGTC	TGTTCATATGTTCCCATGGTATTT	58
5	CCAATTTGTGCGTTTTGGAT	CCAGCAGTAAGATGCAGGTG	63
6	TTCAGGAATCAAACGCAGTACG	TCACTAAATGCGTCGGAGCA	55
7	GTTGACGTGCGGCGTGGGT	CCTTCTTGAGCTTCTGTTGTAATAGC	58
9	CAAAGACAACAAGAAGACTATGAGGA	ACGAATACGAAGATTTCAATAACGC	55
10	CCTGTTCATCGCAGTTGATAGTGTC	GGCGAAGTATGAATATGACGACCGT	58
11	ATGACATCAAATACTGGCCGTTTG	CTGTGCCTCATTTGTCCAACATAC	55
12	CTGGTCAGACTTGTGTGTGGATAATGAT	TAGTAAAGCGAAGTATGAATAGGACCTG	58
14	CGAAGGCGTTTAGGACAAGTG	CGACGAGCGATTTCTTTATGC	55
15	CGTGTATCTCGTCGGTACTGG	GTACTGACATCTTCTCCCAAGGC	55
16	TGGTCTACTTCTGCTTGACGAC	CGCCAGACGGACCAAGTTTC	55
17	TACATTTCATTACAATTTGGTTCGTTTACTACG	TTGGAATCGTAGAGAGCCTGGGTT	58
18	ACGAGGAATATCACGGTTGGAGTTATC	GTGCATGTCTGTTCTCCTAACTCAATC	58
19	CAGGATCCCACTTCATAACGATTG	CAAATCCTTTGAAAGAGCCAACTG	55
20	ATGACTTATTCAGGATGGTGGAGCTC	GATCTGAGCAGCGTGAACATCG	58
21	TCAACTGTTGGCCATTACCT	CGCCAATGCAAGATTTCCACTC	55
22	GTAGCAACTGGCTTCGAGTTG	TGTCACAGGCAAGGTTACAACTG	55
23	GCAGAGTTAATTAAATACAGAATGAGAG	CGAAATATTCCAGCAAGCATCAC	55
24	CACGCTGTTGTACCTGCTGTA	TGCTCTGCTACCTGATATGCC	55
26	ATGGCATATAATCCGGAAGAGACA	CGCTAACTTGTGCACTCTCTAC	55
27	GTGGCAACCAAACAGTGGCTC	TCGCGAAGCTCCGAAGAT	55
28	ATTGCCACAAATTTTCAGCTCGT	GAAGAGCTGGGACACGAGAG	55
29	AATCTTGAATTCCCTGCTATCGC	TAACCTTCATTGTTGCTCAACAATGT	55
30	CGTCCTATTCTCAGAACTTATTCG	CAGAGAACATCTTCGATATACGTAG	55
31	CCTAAACCATCTTCAGGAGTAAAGCATA	AGTCCCATAGTAACAATAGATGAAAAGCTG	55
32	AGATGGAGTCCCGAGAATATCG	AGCAAAGAGCCACAAACACACA	55
34	TTTCGGTATACGACTGCGTTTG	GATCAGTGTCCTTTCTGTGAACATC	55
35	TCATCTCTTGGGACTCGTTGGT	ACTTCCTTTTGAGTTTTCGCATCC	55
36	AGTGTTTTAGCCGAGCACAGGAC	TCTTATCACTCGCATTGGCCTTTC	58
37	GGAGGACATGCAAGTGATTTACG	TTCTATTCCACCGAGCAACTCC	55
38	CTTCAACTACTACGCGTCCATG	CTTCACTATCCCCTTCAAAATTCTCA	55
40	GCCTCGTGTATTTGGCTGATTC	CCTGTGACTTGAGATGCCATTG	55
41	CTGCCTCGCGTCATCTATAG	CCTGTATTACCGGCGTGTTCT	55
42	CTTTCGCCGTCCTAAGTAACG	CAGTCAAGCGCGTAGGTTTAC	55
43	TTCGCGGTTATAGCCCAGAGG	CGACGTGTTGCGGTTGTTGTCT	58
GR4	GCTGGATGAGTTCCTGAGCAA	CAGTTCCTTGGATAGCTGCCT	55

## Results

### Sequencing and identification of OR and IR genes

A total of 464307 reads (average read length 324 bp) were obtained for the male sample and 467771 reads (average read length 328 bp) for the female sample. Assemblies led to the generation of 11007 and 12419 contigs larger than 100 bp, with 6233 and 6589 contigs above 500 bp, in male and female samples, respectively.

**Figure 1:**
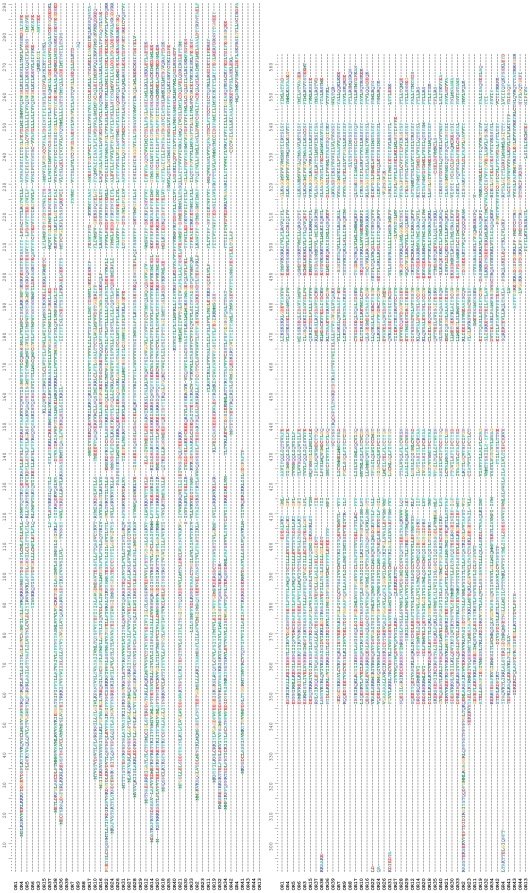
Amino-acid alignment of putative *Cydia pomonella* ORs and GRs.

Bioinformatic analysis led to the identification of a total of 44 different sequences encoding candidate ORs, 29 of which were assembled from both male and female contigs. Of these 44 sequences, 41 have been deposited in the Genbank database under the accession numbers JN836671 to JN836711, while three sequences (CpomOR8, 13 and 44) shorter than 200 bp are given in supplementary material S1. As shown in figure 1, the 41 long sequences possess overlapping regions without identity, confirming that they all represent unigenes. We cannot exclude that the three short sequences may represent the 3′ coding part of non overlapping longer sequences, namely OR5, 11, 23 or 26, thus reducing the total OR unigene number to 41. CpomORs were named according to their similarities with previously annotated Lepidoptera ORs. Sixteen appeared to contain a full length ORF, allowing predictions of transmembrane domains. Depending on the algorithm, CpomORs contained between 4 and 8 transmembrane domains (Table 2), as observed for other insect ORs [3], with 6 domains being the most frequent prediction (37.5%). Topology predictions from TMpred indicated that nine of the sixteen CpomORs may have the N-terminus inside the cell membrane (Table 2), which would be expected for insect ORs.

**Table 2:** Number of transmembrane domains predicted for CpomORs judged to be complete.

CpomOR	HMMTop	TMHMM 2.0	TMpred
2	6	7	6_i_
4	6	5	6_o_
10	7	5	6_o_
12	7	5	8_i_
14	8	6	7_i_
16	8	5	8_i_
18	8	7	8_o_
19	7	6	6_i_
20	8	6	7_i_
21	6	5	6_i_
24	8	6	6_o_
28	6	5	7_o_
31	6	5	6_i_
34	6	6	7_i_
36	8	4	7_o_
38	5	5	7_i_

_i_N-terminus inside.

_o_N-terminus outside.

Apart from a CpomOR sequence that showed high identity with the conserved insect co-receptor, ORco, most CpomORs had low levels of sequence identity with each other and with other Lepidoptera ORs. Five CpomORs were more conserved and showed sequence similarity with previously identified pheromone receptors in other Lepidoptera. Comparison with recently published small CpomOR fragments, proposed to be pheromone receptors (PRs) [25], revealed that we extended two of these and identified three new, previously unknown putative PR sequences. Three of the previous presumed PR fragments were not re-identified by our analysis. However, two of these only differ by four conservative amino acid substitutions, and may represent polymorphisms of the same gene, or be the result from sequencing error.

**Figure 2:**
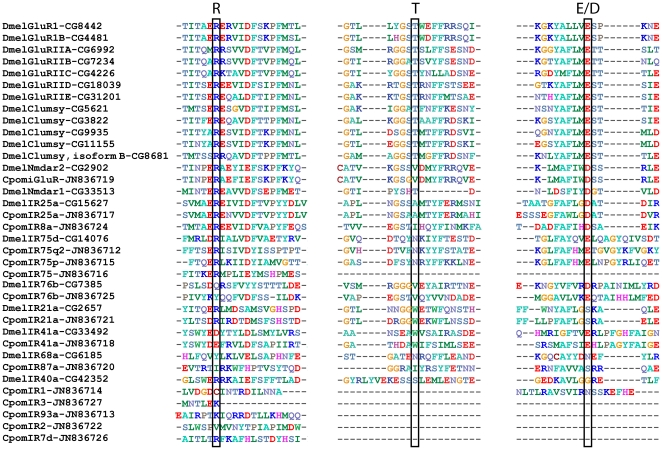
Amino-acid alignment of putative *Cydia pomonella* IRs with *Drosophila melanogaster* IRs and iGlurs. One or more of the three ligand-binding residues critical for iGlur function (bracketed; R, T, E/D) are not conserved in *C. pomonella* IRs, supporting their classification as IRs. Accession numbers for sequences are given in this figure.

One candidate iGluR and 15 candidate IR genes were also identified. These 16 sequences have been deposited in the Genbank database under accession numbers JN836712 to JN836727. Alignment revealed that all 16 *C. pomonella* sequences represent unigenes, since they possess overlapping regions without identity (Fig. 2). *Cydia pomonella* IRs were named according to their similarities with *D. melanogaster* and *B. mori* IRs [17]. One sequence presented similarity with an IR sequence only found in *S. littoralis* [19] and was named CpomIR1, accordingly. Three sequences did not present similarity with already characterized IR encoding genes but retained their characteristic features, and thus were named CpomIR2, 3 and 4. For 13 of the 15 IRs, corresponding contigs were found in both sexes; however, only a male contig was found for CpomIR3, and only a female contig for CpomIR4.

Structure analyses, as well as sequence alignments, showed that the putative full length CpomIRs have a structural organization similar to that of IRs [6], comprising three transmembrane domains, one ion channel pore and a bipartite ligand-binding domain with two lobes (data not shown). Alignment of the predicted binding domains revealed that one or several of the three key amino acids found in iGluR to interact with glutamate (a structural feature used to distinguish between iGluRs and IRs) [6], are not present in CpomIRs that have sequence corresponding to the binding domains (Fig. 2). Four of the IRs appeared to contain a full length ORF (CpomI25a, 41a, 75q2, and 76b). TMHMM2.0, TMpred and HMMtop predicted three or more transmembrane domains for all of these (Table 3), as would be expected for IRs.

**Table 3:** Number of transmembrane domains predicted for CpomIRs judged to be complete.

CpomIR	HMMTop	TMHMM 2.0	TMpred
25a	3	3	5_o_
41a	3	3	4_o_
75q2	3	3	4_i_
76b	4	3	7_o_

_i_N-terminus inside.

_o_N-terminus outside.

### Sequence similarity analysis

**Figure 3:**
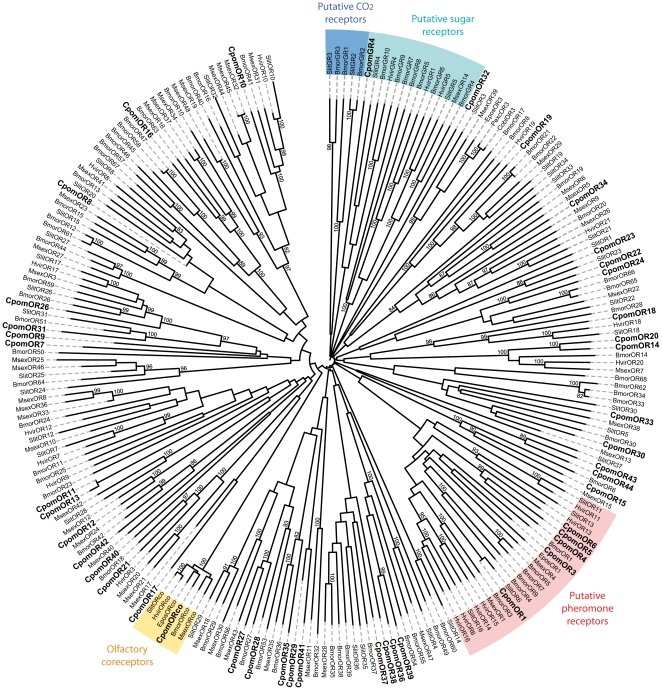
Neighbor-joining tree of candidate odorant (OR) and gustatory (GR) receptor genes from *Cydia pomonella* and other Lepidoptera. The tree was drawn with iTOL, based on an unrooted tree constructed using the BioNJ algorithm in Seaview v.4, which was made based on a sequence alignment using MAFFT version 6. Cpom, *C. pomonella* (this paper), Bmor, *Bombyx mori* [61], Cobl, Ctenopseustis obliquana [33], Epos, Epiphyas postvittana [33], Hvir, *Heliothis virescens* [50], [56], Msex, *Manduca sexta* [15], Pexc, Planotortrix excessana [33], Slit, *Spodoptera littoralis* [16; Jacquin–Joly, unpublished data].

The annotation of five ORs as candidate CpomPRs (CpomOR1, 3, 4, 5, and 6) was confirmed by sequence similarity analysis (Fig. 3), as they all clustered within the conserved clade containing functionally characterized Lepidoptera pheromone receptors [29], [30], [31], [32]. Within this clade, CpomOR3 was sister-group (albeit with low bootstrap support) to EposOR1 from the tortricid moth *Epiphyas postvittana*, characterized as a plant volatile receptor rather than a sex pheromone receptor [33]. As expected, the CpomOR sequence showing high identity with the conserved insect co-receptor clustered in the ORco clade. At least one Lepidoptera ortholog could be assigned to the majority of the putative CpomORs, but nine of them had no counterpart (CpomOR7, 9, 11, 13, 29, 32, 41, 43, and 44). Intriguingly, none of the CpomORs clustered with EposOR3, CoblOR3 and PtorOR3, identified in other tortricid moths [33]. A homolog of the *B. mori* female-enriched receptor BmorOR30 was found (CpomOR30), but no homologs of the other *B. mori* female-enriched receptors BmorOR19, 45, 46, 47 and 50 [34], [35] could be identified. One of the putative ORs, CpomOR25, clustered with candidate GRs proposed to be sugar receptors [36], and was thus reclassified as a GR and renamed CpomGR4.

**Figure 4:**
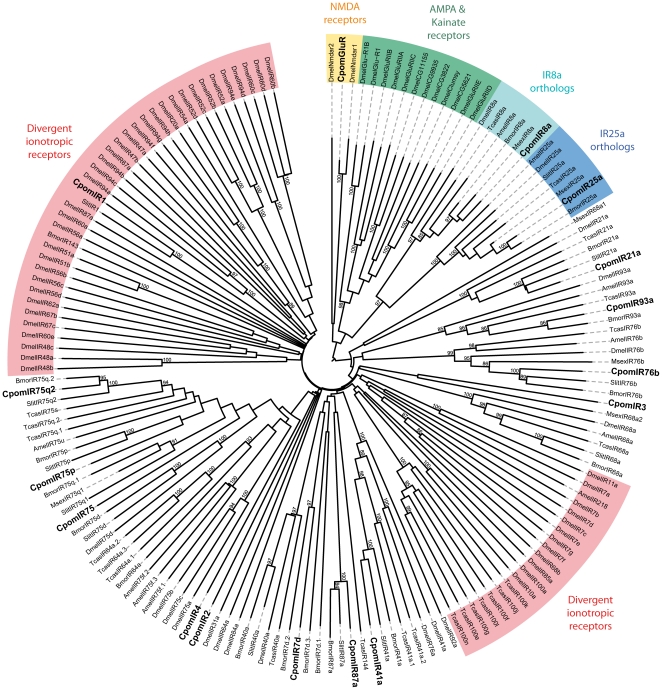
Neighbor-joining tree for candidate ionotropic receptor (IR) genes from *Cydia pomonella* and other insects. The tree was drawn with iTOL, based on an unrooted tree constructed using the BioNJ algorithm in Seaview v.4, which was made based on a sequence alignment using MAFFT version 6. Cpom, *C. pomonella* (this paper), Amel, *Apis mellifera* [17], Bmor, *Bombyx mori* [17], Dmel, *Drosophila melanogaster* [6], Msex, *Manduca sexta* [15], Slit, *Spodoptera littoralis* [16], Tcas, *Tribolium castaneum* [17].

In the IR neighbor-joining tree (Fig. 4), CpomIRs did not cluster with insect iGlurs, confirming their annotation as IRs. CpomIR1 clustered – together with its ortholog from *S. littoralis* – in a “divergent IR” clade but without any bootstrap support, so we can not infer any evolutionary relationship between CpomIR1 and these divergent IRs. As expected, two CpomIRs clustered in the highly conserved IR8a and IR25a sub-families (Fig. 4). At least one insect IR ortholog could be assigned to the majority of the putative CpomIRs, but three of them have no counterpart (CpomIR2, 3 and 4). Functional studies of IRs are limited to a handful of *D. melanogaster* IRs [6], [37], but none of the CpomIRs clustered closely with one of these. The exception is CpomIR76b, which is closely related to *D. melanogaster* IR76b that, when expressed together with the co-receptor DmelIR25a and DmelIR76a, confers reception of phenylethyl amine [18].

### RT-PCR for expression analysis

**Figure 5:**
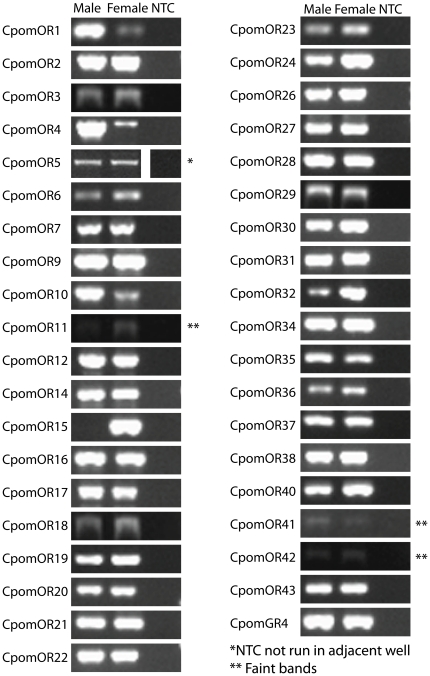
Sex specific expression of *Cydia pomonella* OR & GR genes. Gel electrophoresis of RT-PCR products using antennal RNAs from male and female *C. pomonella*, with primers designed to amplify putative CpomOR & GR genes. NTC, No Template Control.

Out of the 44 OR and GR sequences, 40 sequences were long enough to enable the design of primers giving a product of 300 bp or more, while four were too short (CpomORs 8, 13, 39 and 44). For these 40 genes, expression in male and female antennae was tested using RT-PCR (Fig. 5). Of these, 38 were found to be expressed in the antennae of both sexes (including CpomGR4). In 11 cases (CpomORs 1, 4, 5, 6, 9, 17, 23, 26, 32, 35, and 43), expression was found in both sexes, although a corresponding contig was found only in one sex. One putative OR, CpomOR15, was found to be female-specific. Sequencing confirmed the identity of all these products. For three of the predicted ORs (CpomORs 11, 41 and 42), RT-PCR on antennal cDNAs gave faint bands of correct size, which could not be verified by sequencing. CpomOR33 gave no product in either sex, despite using two sets of primers designed to amplify different parts of the corresponding contig.

## Discussion

We have identified 43 candidate OR gene sequences, that may represent 40 to 43 unigenes, one GR, 15 IR and one iGluR unigene in the codling moth, *C. pomonella*. This is the first comprehensive study of chemosensory receptors in a moth of the tortricid family, which includes numerous species of economic importance in agriculture, horticulture and forestry. Our transcriptomic strategy appeared to be very fruitful in identifying large sets of chemosensory receptors from different sub-families. For comparison, *S. littoralis* male antennal transcriptome sequencing led to the identification of only 29 ORs, 2 GRs and 12 IRs [16], [19], and in *M. sexta*, next-generation sequencing of both male and female antennae led to the identification of 47 ORs but only 6 IRs [15].

### OR and GR identification in *C. pomonella* antennal transcriptome

Previous studies have suggested that the insect olfactory system follows an organization where a single OSN class expresses, apart from ORco, a single OR [38], with some exceptions [39], [40]. In turn, each OSN type innervates a single glomerulus in the antennal lobe, the primary olfactory center in the insect brain [38]. While the relationship is not exactly 1∶1∶1, e.g. due to the presence of other classes of chemoreceptors (such as ionotropic receptors and gustatory receptors), the number of glomeruli in a species should give a rough approximation of how many ORs are present [15], [41]. A previous study found 50±2 glomeruli in *C. pomonella* males, and 49±2 in females [42], and our findings thus agree well with the number of ORs that would be expected to be expressed, taking into account that some glomeruli should be innervated by OSNs expressing either IRs or GRs.

In the sequence similarity analysis of the *C. pomonella* ORs, five of them grouped in a conserved clade containing lepidopteran PRs (Fig. 3), and we thus hypothesize that some or all of them are involved in pheromone reception. Among those five receptors, CpomOR3 may be related to EposOR1 from the light brown apple moth *E. postvittana*, but the bootstrap value for this node was low, probably due to the short length of the CpomOR3 sequence. EposOR1 is of particular interest, because it did not respond to pheromone compounds when expressed in Sf9 cells but was highly sensitive to methyl salicylate [33], which elicits strong antennal responses in *C. pomonella* [43]. Six pheromone compounds are known in *C. pomonella* [44], [45], [46], [47], and four classes of OSNs with partially overlapping detection ranges have been found to be involved in their detection [24], [48], [49]. While the pheromone seems to be attractive only to males, both sexes have been found to have pheromone-detecting OSNs [42], [48], suggesting that both sexes would express PRs in their antennae. In accordance with this, results from the RT-PCR analysis indicated that all putative *C. pomonella* pheromone receptors are expressed in the antennae of both sexes. Although PR expression in most Lepidoptera has been shown to be restricted to male antennae [11], [29], [50], two candidate PRs identified in *S. littoralis* were found to be expressed in antennae of both sexes [16], fitting well with the observation that *S. littoralis* females, like *C. pomonella* females, detect their own pheromone [51]. The rationale behind female pheromone perception has been proposed to be optimization of pheromone production and spatial dispersion of females over host plants [52], [53].

Excluding the five CpomORs that we were not able to study by RT-PCR, all CpomORs were found to be expressed in the antennae of both sexes, except CpomOR15, which was female-specific (Fig. 5). Its closest homologs are BmorOR6 and MsexOR15, neither of which has been functionally characterized. BmorOR6 has been shown to have a male bias in antennal expression, however, and has thus been proposed to be a PR in *B. mori* [34]. Up to now, functional proof of this classification is lacking, and BmorOR6 and its orthologs are usually excluded from the conserved PR clade.

In the OR tree (Fig. 3), one CpomOR grouped close to the OR18 conserved receptor family recently proposed to be specific to noctuids [14]. However, it exhibited less than 50% sequence identity with noctuid OR18 sequences, whereas OR18 present an average of 88% identity within noctuids. Thus, there is no obvious conservation of this gene between tortricids and noctuids [34], [35].

The gustatory receptor we identified, CpomGR4, was found in a clade with sugar receptors (Fig. 3), which included the newly characterized *B. mori* fructose receptor (BmorGR9) [54] and inositol receptor (BmorGR8) [55]. Other chemosensory receptors identified in moth antennae also clustered in this family (e.g. SlitGR4 and 5, and HvirGR1, 4, and 5) [16], [56], in concordance with electrophysiological results indicating that moth antennae, in addition to the proboscis, are involved in sugar detection [57]. Sugars and other carbohydrates have been shown to influence host preference and oviposition in codling moth females [58].

### IR identification in *C. pomonella* antennal transcriptome

Up to now, only two studies reported IR expression in Lepidoptera antennae [15], [19]. Here, we extend IR transcript identification in antennae in this insect order. The number of IRs found in *C. pomonella* (15) is similar to that found in *B. mori* and *S. littoralis* [17], [19], and includes two candidate genes homologous to the co-receptors IR8a and IR25a [18]. As IRs have more complicated expression patterns than ORs, with 2–5 IRs expressed in a single OSN [6], it is harder to predict the number of glomeruli in the antennal lobe they should innervate. For instance, the closest homolog of CpomIR76b, DmelIR76b (Fig. 4), requires the expression of DmelIR76a as well as the co-receptor DmelIR25a for correct reception of the ligand phenylethyl amine [18]. CpomIR76b is the only CpomIR for which a homolog has been functionally characterized, but it is not known if *C. pomonella* antennae detect phenylethyl amine. A structurally related compound, 2-phenylethanol, which is produced by flowers [59] and also ripe apples [43], is detected by *C. pomonella* and other moths [43], [60].

Two subfamilies of IRs have been recently distinguished: the conserved “antennal IRs” and the species-specific “divergent IRs” [17]. Ten of the CpomIRs we identified belong to the antennal IR subfamily, a number similar to that found in, *e.g., B. mori* (11) and *S. littoralis* (10) [17], [19], suggesting that we may have established the entire repertoire of antennal IRs in *C. pomonella*. A new Lepidoptera subtype of antennal IRs (IR87a) was recently proposed based on specific expression in antennae [19]. Supporting this view, an IR87a homolog (clustered with SlitIR87a and BmorIR87a in the neighbor-joining tree) was identified in *C. pomonella* antennae. We also found a homolog to the previously identified SlitIR1, which was initially proposed to be a unique divergent sequence among insects [19]. While no *B. mori* ortholog clusters with the two sequences, the identification of a member of this lineage in Tortricidae means that, unlike previously believed, it is not restricted to Noctuids [19]. Notably, we identified three new IR subtypes expressed in *C. pomonella* antennae (CpomIR2, 3 and 4) that had no *B. mori* ortholog. Further IR identification in other Lepidoptera families would reveal when these new IR subtypes arose.

### Conclusion

Our approach has been successful in identifying what appears to be a large part of the OR and IR repertoires in a non-model pest species. This enables further investigation of chemosensation in the codling moth, in particular regarding sex pheromone detection. The discovery of ORs and IRs will also assist in the identification of novel volatile host compounds, which would give new options for control by disruption, mass trapping, or trap crops.

## Supporting Information

Supplementary Material S1 Fasta of CpomORs not submitted to Genbank (short sequences).
(DOC)
