# Supplementary material for: Putative Chemosensory Receptors of the Codling Moth, Cydia pomonella, Identified by Antennal Transcriptome Analysis
Source: PLoS One. 2012 Feb 20;7(2):e31620. doi: 10.1371/journal.pone.0031620 (PMC3282773; doi:10.1371/journal.pone.0031620)
Supplement: Supplementary Material S1 — Fasta of CpomORs not submitted to Genbank (short sequences). (DOC) [file pone.0031620.s001.doc]

>CpomOR8 mRNA, partial cds

TCGCTTACCAGTTTCACTTCTATTATGAATTCTTCGTACTCATTCTTCACTTTGCTGCGTCACATGCAATCTCGCCAAAATTAG

>CpomOR13 mRNA, partial cds

CCAATATTTATTTTAAAGGTTATACGATCGCGGGCGACGTTATTGTCAATCTCGTTGATAAAATGTATGACAGAATTTAACATACCAATCGTCAATTAG

>CpomOR44 mRNA, partial cds

TTAGTAGGTGGCAAACGGGCAGACGGATCACCTGATGGTAAGCGATTACCGCCGCCCATGGACACTTGCAACACTAGAGGGGACACAAATAAATGCCCTTACCGGGATTCGAATCACTACCCACTAGGCCAGACCGGTCATCCTATATAA

>CpomOR8

SLTSFTSIMNSSYSFFTLLRHMQSRQN*

>CpomOR13

PIFILKVIRSRATLLSISLIKCMTEFNIPIVN*

>CpomOR44

LVGGKRADGSPDGKRLPPPMDTCNTRGDTNKCPYRDSNHYPLGQTGHPI*
